# Supplementary figures and images for: Genotyping Analysis of Cryptococcus deuterogattii and Correlation with Virulence Factors and Antifungal Susceptibility by the Clinical and Laboratory Standards Institute and the European Committee on Antifungal Susceptibility Testing Methods
Source: J Fungi (Basel). 2023 Aug 31;9(9):889. doi: 10.3390/jof9090889 (PMC10532325; doi:10.3390/jof9090889)

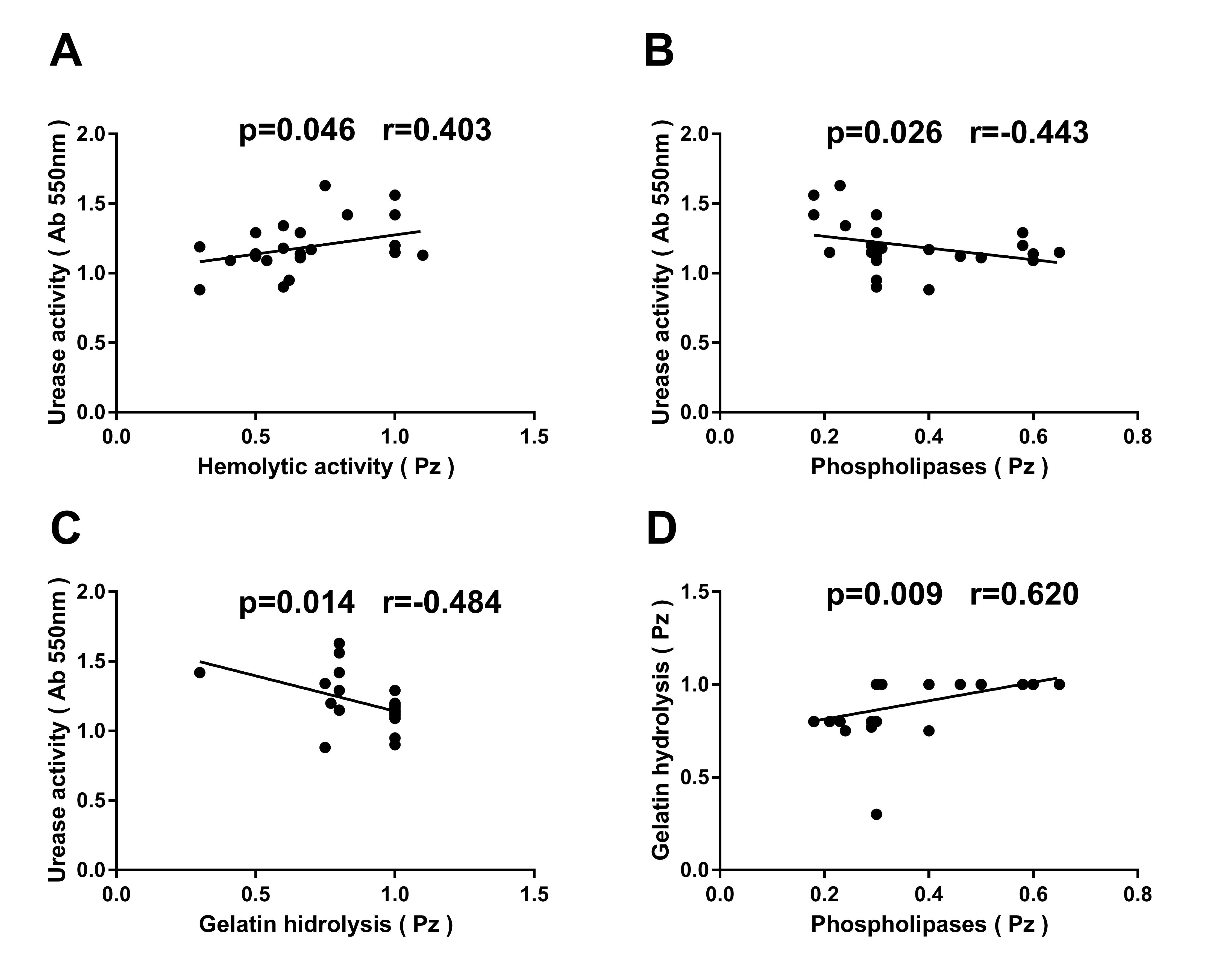

Supplement: Supplementary file 1 [file jof-09-00889-s001.zip › Figure S1.tif]
